# Supplementary material for: Ubiquitin transfer by a RING E3 ligase occurs from a closed E2~ubiquitin conformation
Source: Nat Commun. 2020 Jun 5;11:2846. doi: 10.1038/s41467-020-16666-y (PMC7275055; doi:10.1038/s41467-020-16666-y)
Supplement: Supplementary file 1 — Supplementary Information [file 41467_2020_16666_MOESM1_ESM.pdf]

Supplementary Information

Ubiquitin transfer by a RING E3 ligase occurs from a closed E2~ubiquitin conformation

Emma Branigan *et al.*

Supplementary Figure 1

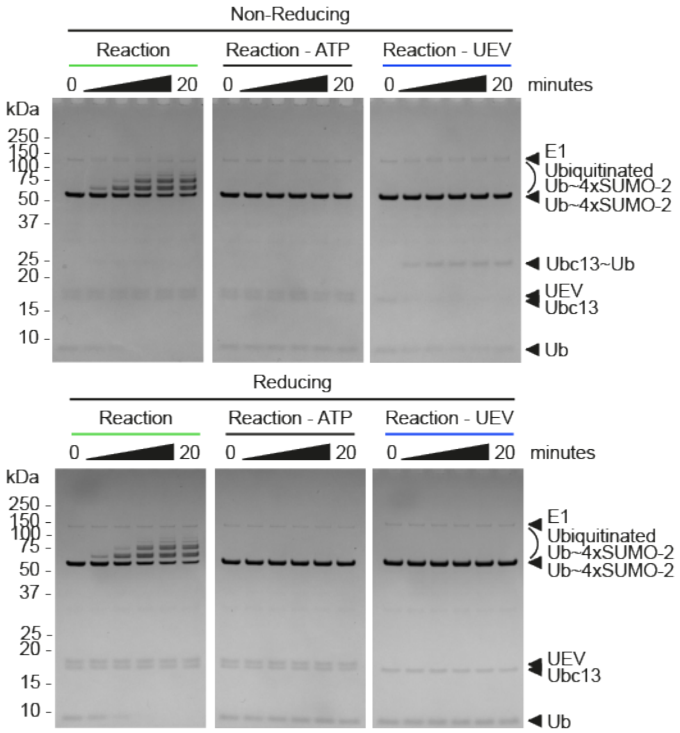

Supplementary Fig. 1 SDS-PAGE analysis of fluorescence polarization ubiquitination assays. Coomassie stained images of SDS-PAGE analysis of fluorescence polarization ubiquitination assays for the Reaction (green), Reaction - ATP (black) and Reaction - UEV (blue) shown in **Fig. 1 c and d**, under non-reducing (above) and reducing (below) conditions. Source data are provided as a Source Data file.

Supplementary Figure 2

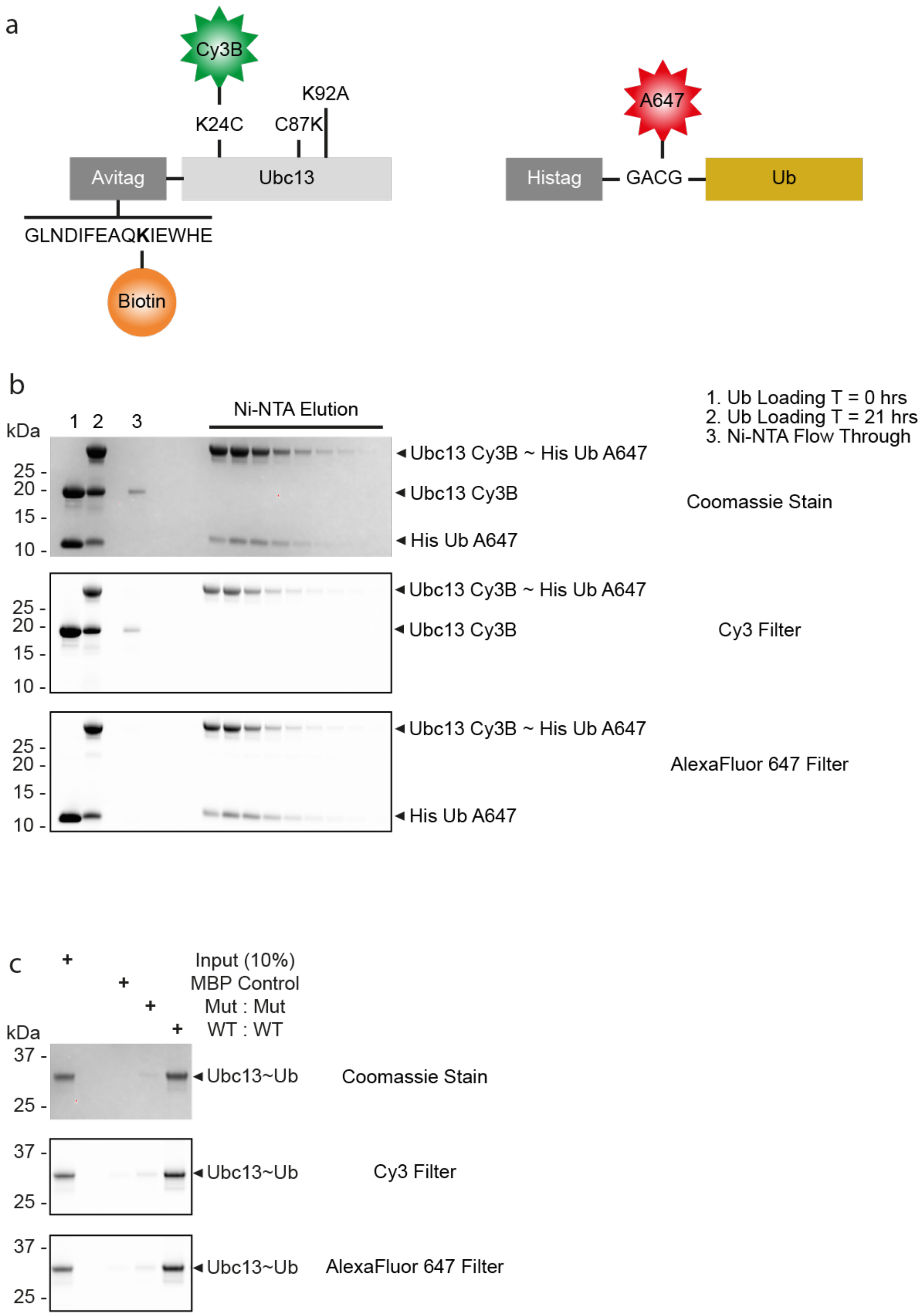

Supplementary Fig. 2 Production, purification and validation of an isopeptide linked and FRET labelled Ubc13~Ub conjugate. **a** Schematic diagrams of the Ubc13 and

ubiquitin constructs used in FRET analysis of a stable isopeptide linked Ubc13~Ub conjugate. The Ubc13 construct contains a biotinylated (orange) N-terminal avitag (dark grey) followed by a short linker and Ubc13 (light grey), where Ubc13 contains the mutations K24C, C87K and K92A. Ubc13 is labelled with Cy3B (green) at position K24C. The ubiquitin construct contains an N-terminal histag (dark grey) and a short linker containing a cysteine for labelling with AlexaFluor 647 (red), followed by ubiquitin (yellow). **b** SDS-PAGE analysis showing generation of the stable isopeptide linked Ubc13~Ub conjugate and purification by Ni-NTA chromatography. **c** RNF4 RING domain dimer pull-down experiment showing binding to the FRET labelled isopeptide linked Ubc13~Ub conjugate, analysed by SDS-PAGE. WT denotes a wild-type RING domain, while Mut denotes a RING domain containing E2~Ub binding site mutations (M140A and R181A). For **b** and **c**, gels are imaged using Cy3 and AlexaFluor 647 filters, followed by Coomassie staining and imaging using a Coomassie filter. Source data are provided as a Source Data file.

Supplementary Figure 3

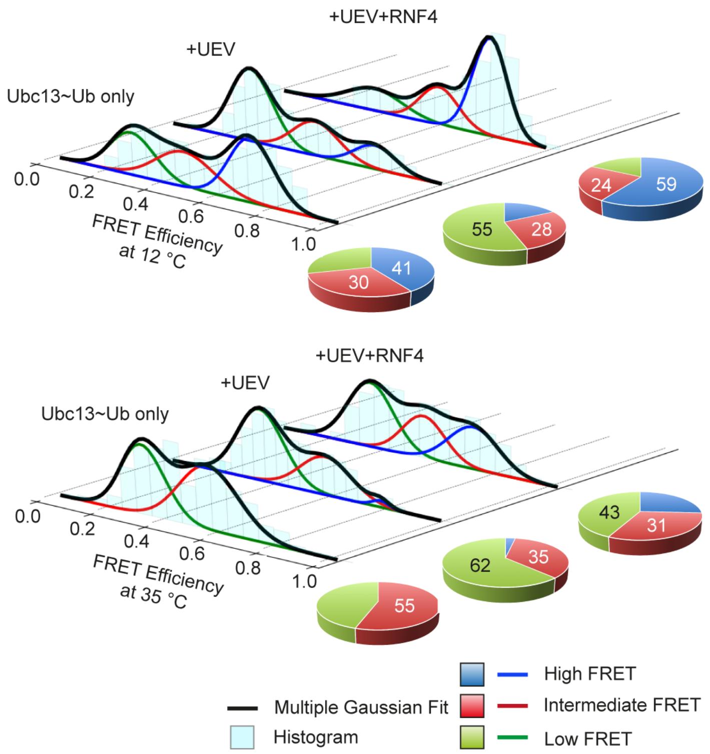

Supplementary Fig. 3 smFRET analysis of the E2~Ub conjugate at 12 and 35 °C. smFRET histograms (cyan) showing the isopeptide linked E2~Ub conjugate conformation alone and in complex with UEV and the RNF4 RING domain dimer at 12 (above) and 35 (below) °C. Gaussians are fitted to the low FRET state (green), the intermediate FRET state (red) and the high FRET state (blue). The multiple Gaussian fit is shown in black. The pie charts to the right of each histogram show the percentage contribution of each FRET state to the overall population, using the same colour scheme as the Gaussians fitted to the smFRET histograms. Source data are provided as a Source Data file.

Supplementary Figure 4

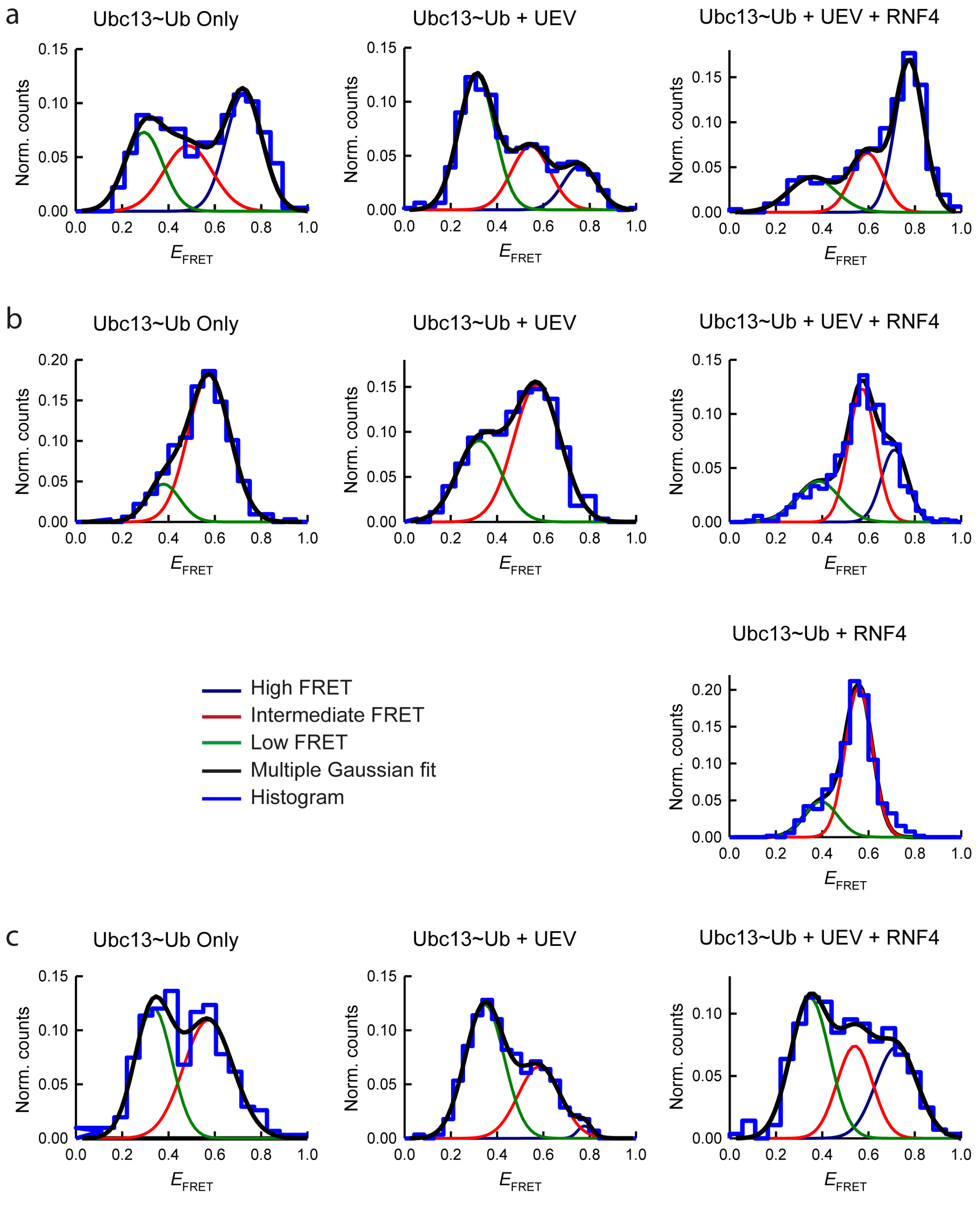

Supplementary Fig. 4 smFRET histograms. **a** Normalized smFRET histograms showing the E2~Ub conjugate conformation alone and in complex with UEV and the RNF4 RING domain dimer at 12 °C. **b** Same as **a** but at 22 °C. The E2~Ub conjugate conformation in the presence of the RNF4 RING domain dimer only is also shown at 22 °C. **c** Same as **a** but at 35 °C. For **a**, **b** and **c** the histogram is outlined in blue.

Gaussians are fitted to the low FRET state (green), the intermediate FRET state (red) and the high FRET state (dark blue). The multiple Gaussian fit is shown in black. Source data are provided as a Source Data file.

Supplementary Figure 5

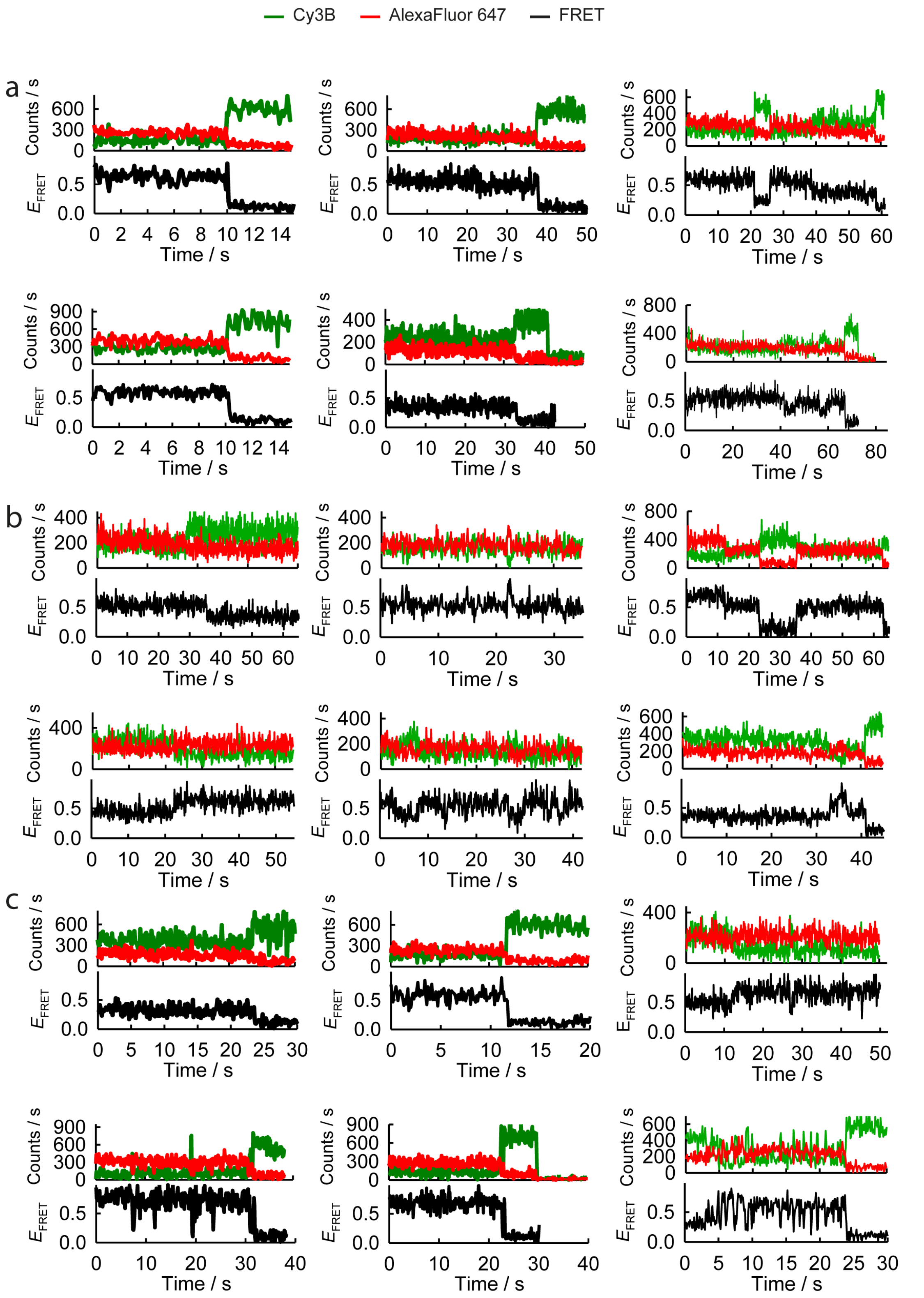

Supplementary Fig. 5 Representative single molecule traces. **a** Representative single molecule traces for the E2~Ub conjugate alone. **b** Same as in **a** but for the E2~Ub

conjugate in complex with UEV. **c** Same as in **a** but for the E2~Ub conjugate in complex with UEV and RNF4 RING domain dimer. For **a**, **b** and **c** each molecule contains a Cy3B (green), AlexaFluor 647 (red) and FRET intensity trace (black). Source data are provided as a Source Data file.

Supplementary Figure 6

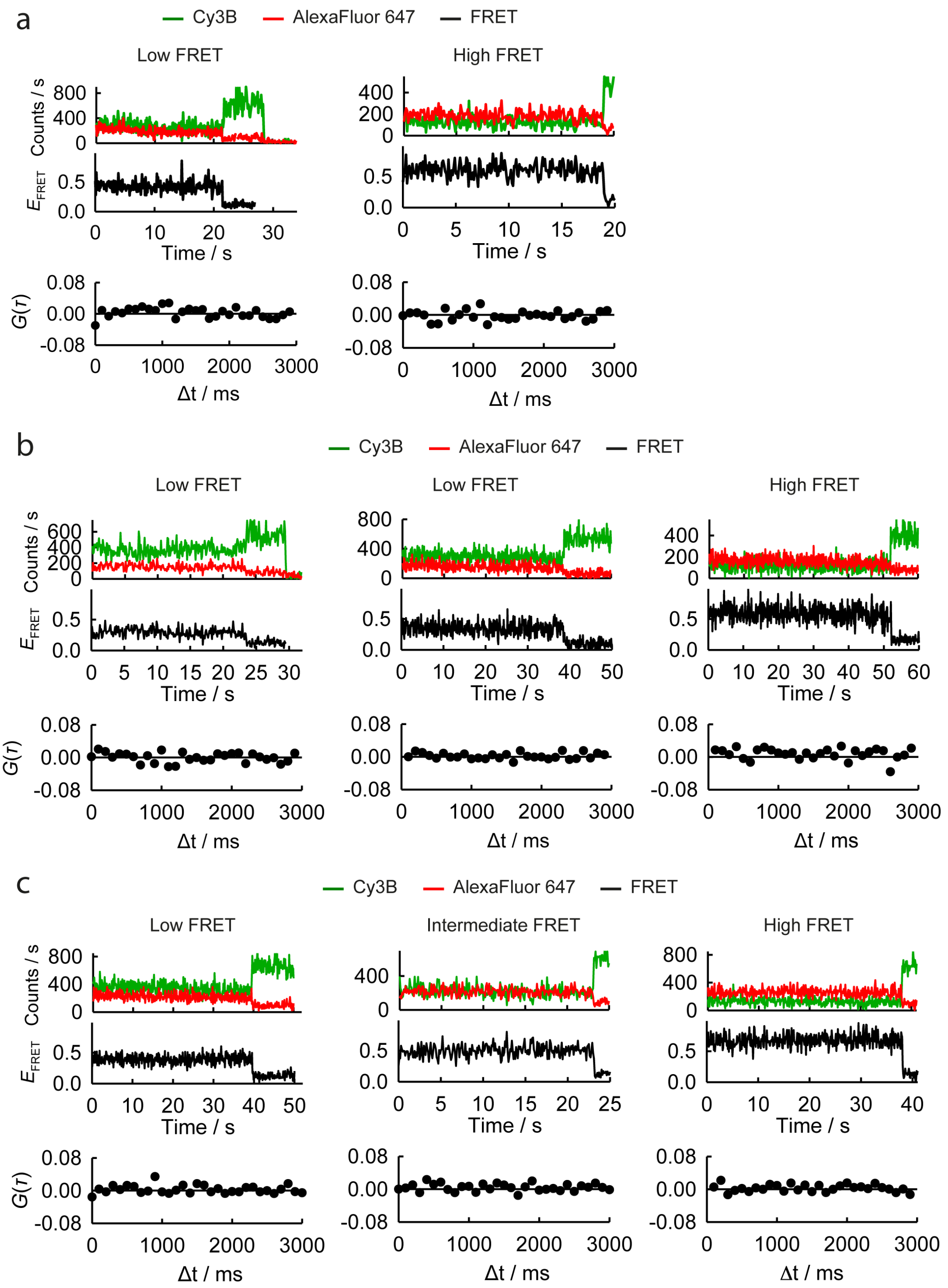

Supplementary Fig. 6 Cross-correlation analysis. **a** Cross-correlation analysis performed on smFRET traces demonstrating the stability of the low, intermediate and high FRET states for the E2~Ub conjugate alone. Each molecule contains a Cy3B

(green), AlexaFluor 647 (red) and FRET intensity trace (black) and a cross-correlation plot below (black cirlces). **b** Same as in **a** but for the E2~Ub conjugate in complex with UEV. **c** Same as in **a** but for the E2~Ub conjugate in complex with UEV and RNF4 RING domain dimer. Source data are provided as a Source Data file.

Supplementary Figure 7

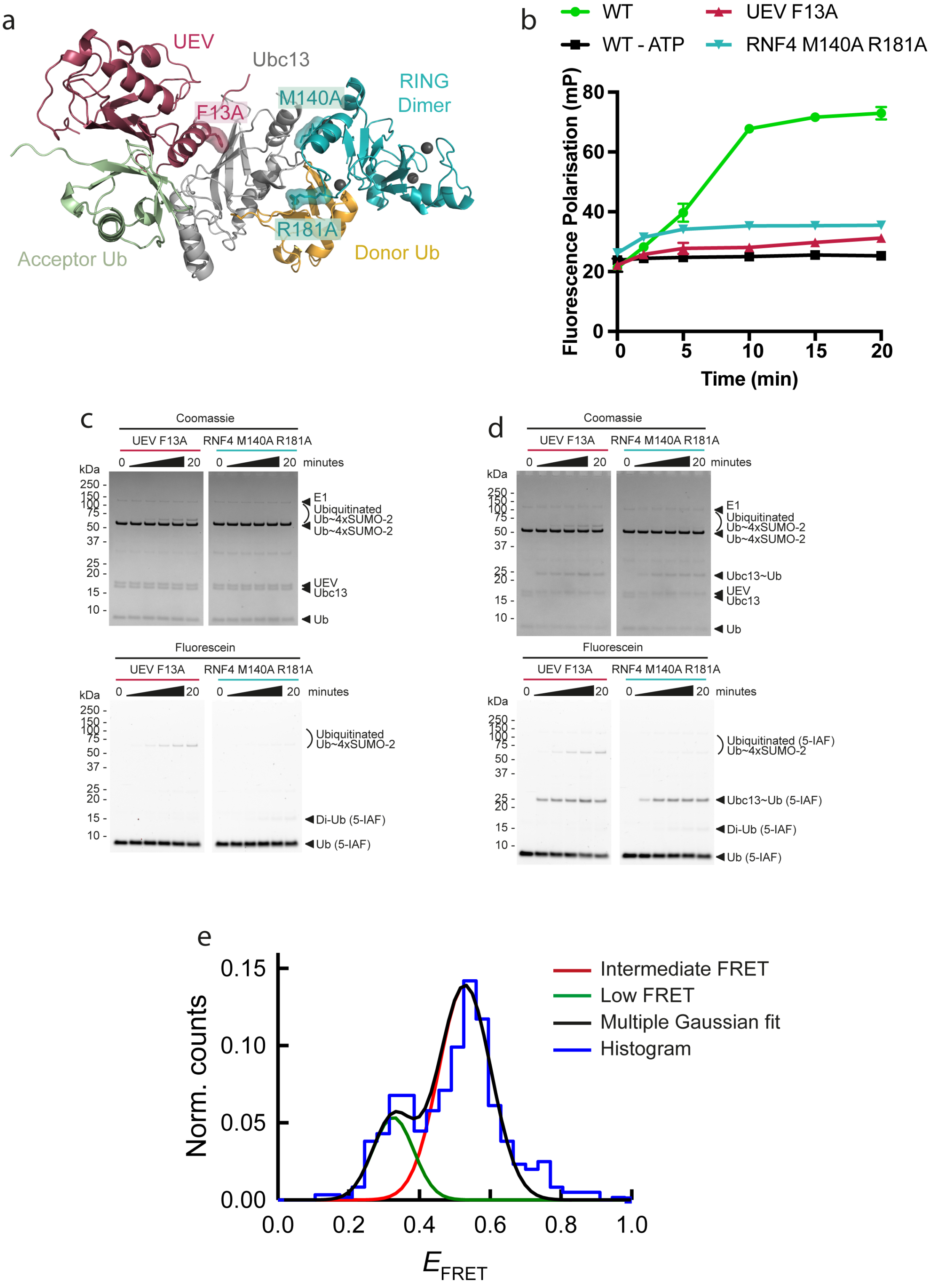

Supplementary Fig. 7 Effect of negative control complex mutations on the conformational state of the E2~Ub conjugate. **a** Locations of mutations made within the complex (PDB accession number 5AIT). **b** Fluorescence polarization

ubiquitination assay showing the effect of mutations on K63-linked polyubiquitin chain formation. Data points represent the mean of n=3 independent experiments and error bars represent ± s.d.. Data points are represented for the reaction containing UEV F13A by raspberry triangles and RNF4 M140A R181A by cyan inverted triangles, with a line drawn in the same colour connecting each data point in a reaction. Error bars are omitted when the error is smaller than the data point. **c** Coomassie stained (above) and fluorescein imaging (below) of SDS-PAGE analysis of fluorescence polarization ubiquitination assays shown in **b**, under reducing conditions. **d** Same as in **c** but under non-reducing conditions. For **c** and **d**, the same colour scheme is used as in **b**. **e** Normalized smFRET histogram showing the E2~Ub conjugate conformation in the presence of UEV F13A and the RNF4 RING domain dimer containing M140A and R181A mutations. The histogram is outlined in blue. Gaussians are fitted to the low FRET state (green) and the intermediate FRET state (red). The multiple Gaussian fit is shown in black. Source data are provided as a Source Data file.

Supplementary Figure 8

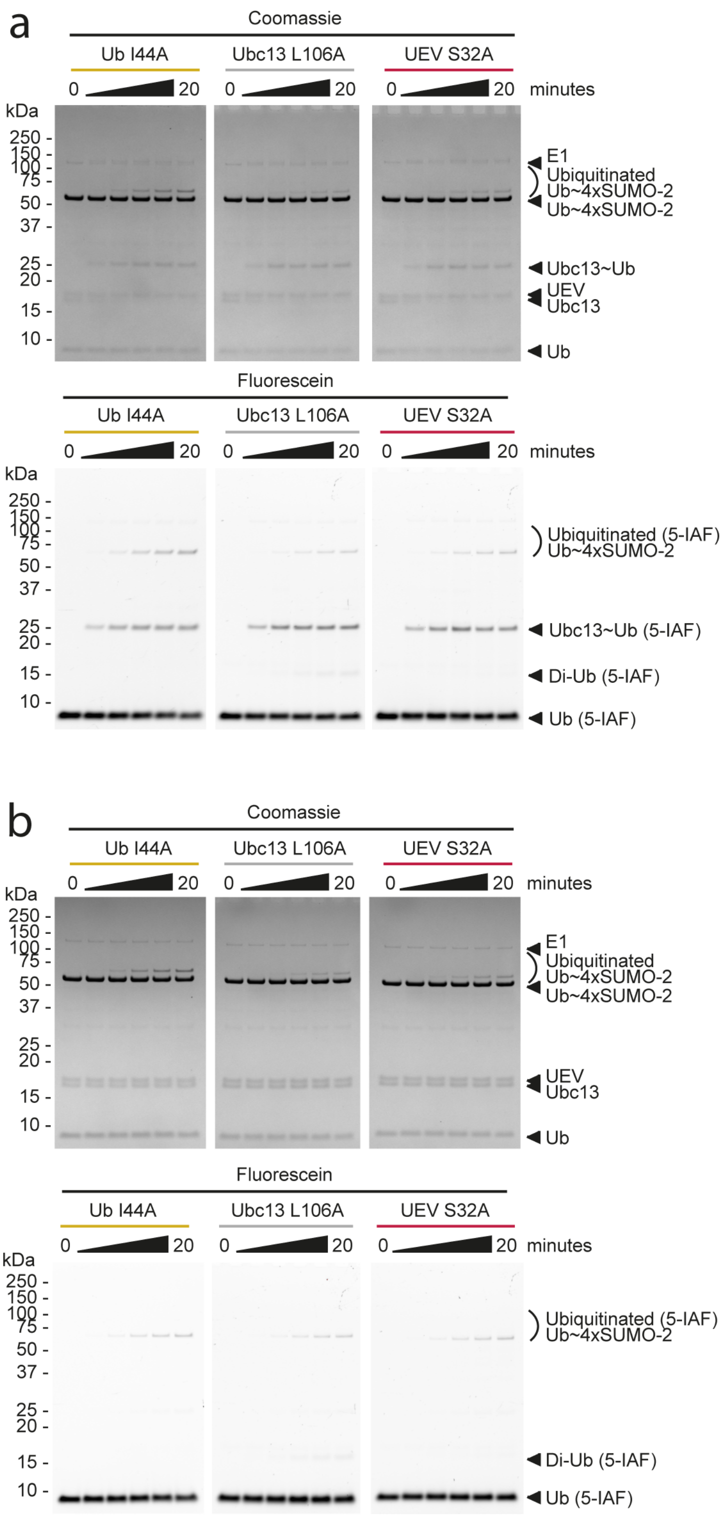

Supplementary Fig. 8 SDS-PAGE analysis of fluorescence polarization ubiquitination assays. **a** Coomassie stained (above) and fluorescein imaging (below) of SDS-PAGE analysis of fluorescence polarization ubiquitination assays containing

Ub I44A (yellow), Ubc13 L106A (grey) and UEV S32A (raspberry) shown in **Fig. 3b**, under non-reducing conditions. **b** Same as in **a** but under reducing conditions. Source data are provided as a Source Data file.

Supplementary Figure 9

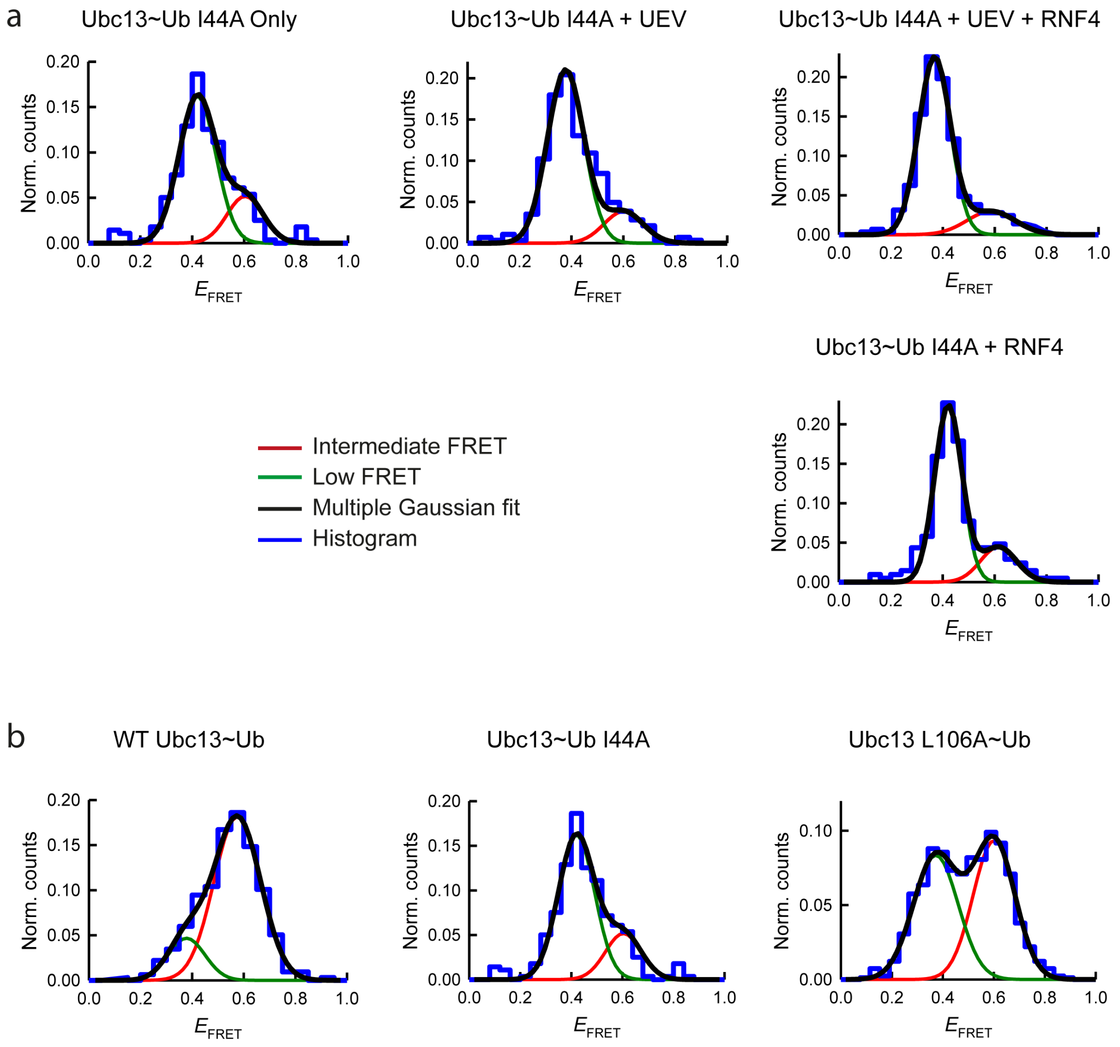

Supplementary Fig. 9 smFRET histograms for Ub I44A and Ubc13 L106A containing Ubc13~Ub conjugates. **a** Normalized smFRET histograms showing the E2~Ub conjugate conformation containing Ub I44A alone and in complex with UEV and the RNF4 RING domain dimer and in the presence of the RNF4 RING domain dimer only. **b** Normalized smFRET histograms showing the conformation of the WT E2~Ub conjugate and the E2~Ub conjugate containing either Ub I44A or Ubc13 L106A. For **a** and **b**, the histogram is outlined in blue. Gaussians are fitted to the low FRET state (green) and the intermediate FRET state (red). The multiple Gaussian fit is shown in black. Source data are provided as a Source Data file.

Supplementary Figure 10

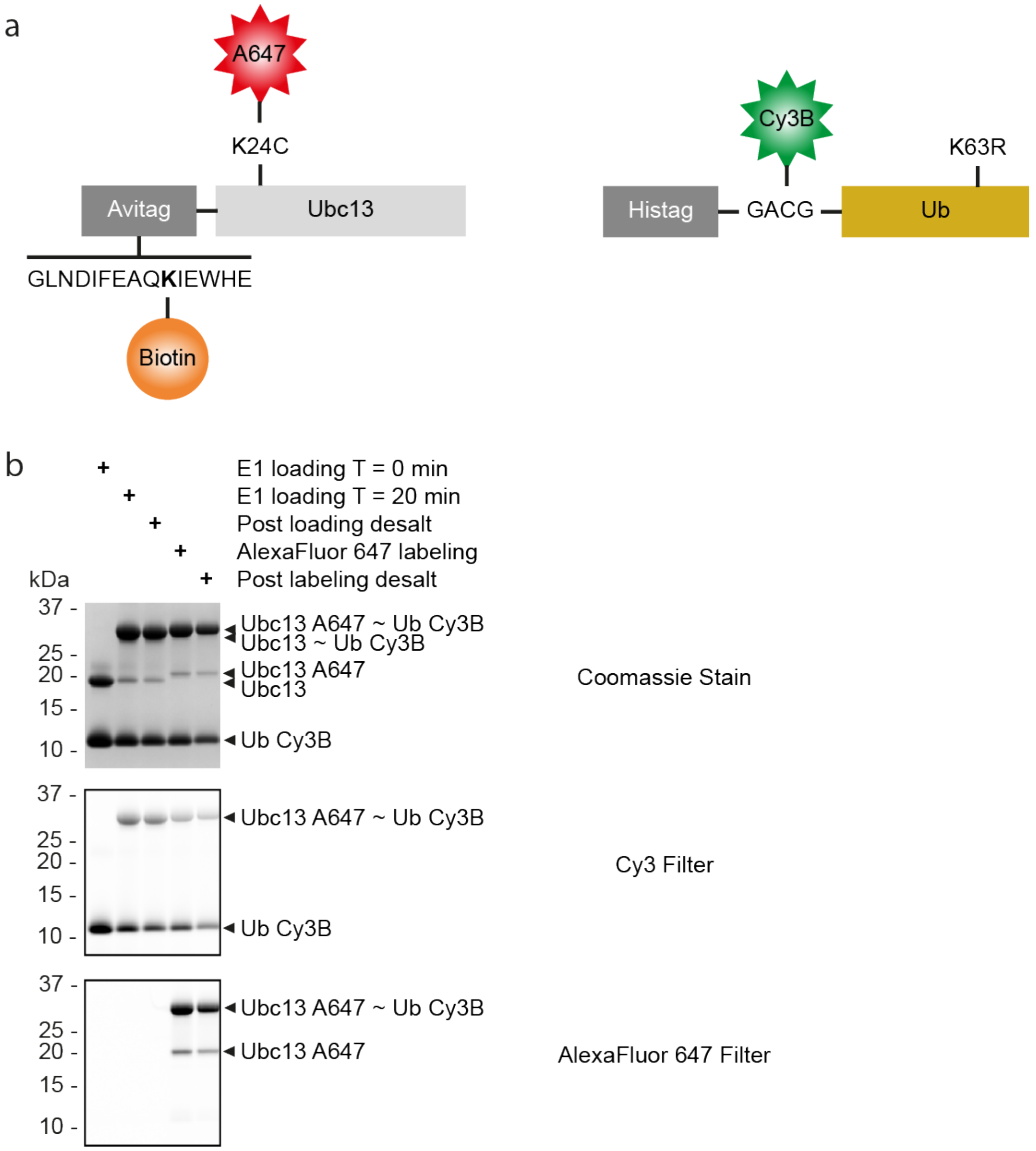

Supplementary Fig. 10 Production and labelling of a thioester linked and FRET labelled Ubc13~Ub conjugate. **a** Schematic diagrams of the Ubc13 and ubiquitin constructs used in FRET analysis of an unstable thioester linked Ubc13~Ub conjugate. The Ubc13 construct contains a biotinylated (orange) N-terminal avitag (dark grey) followed by a short linker and Ubc13 (light grey). Ubc13 contains the mutation K24C that is used for labelling with AlexaFluor 647 (red). The ubiquitin

construct contains an N-terminal histag (dark grey) and a short linker containing a cysteine for labelling with Cy3B. This is followed by ubiquitin (yellow) containing a K63R mutation. **b** SDS-PAGE analysis showing generation of the unstable thioester linked Ubc13~Ub conjugate and labelling with AlexaFluor 647. Gels are imaged using Cy3 and AlexaFluor 647 filters, followed by Coomassie staining and imaging using a Coomassie filter. Source data are provided as a Source Data file.

Supplementary Figure 11

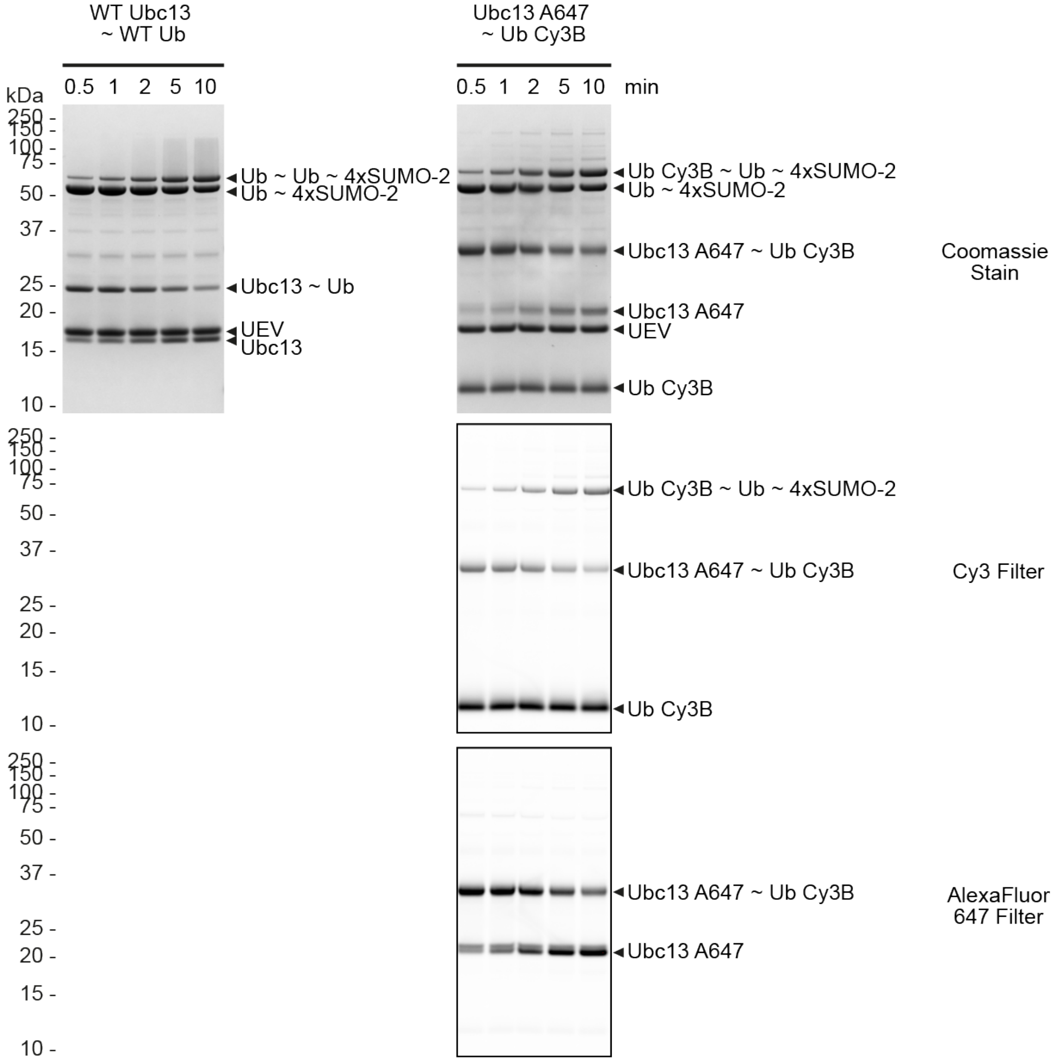

Supplementary Fig. 11 Validation of a thioester linked and FRET labelled Ubc13~Ub conjugate. SDS-PAGE analysis of a substrate single turnover ubiquitination assay showing the ability of a WT Ubc13~Ub compared to the tagged and FRET labelled Ubc13~Ub conjugate to monoubiquitinate the Ub~4xSUMO-2 substrate. Gels are imaged using Cy3 and AlexaFluor 647 filters, followed by Coomassie staining and imaging using a Coomassie filter. Source data are provided as a Source Data file.

Supplementary Figure 12

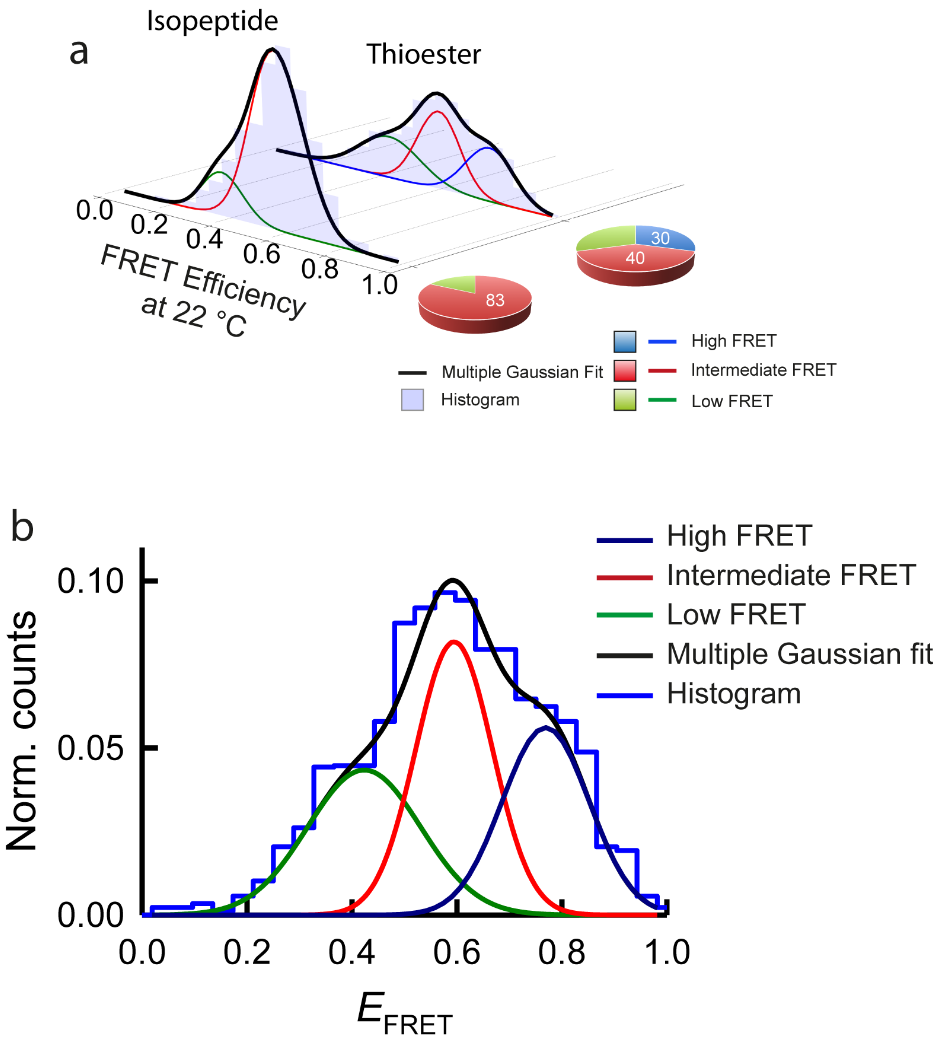

Supplementary Fig. 12 smFRET analysis of the thioester linked E2~Ub conjugate. **a** smFRET histograms (blue) showing the conformation of the stable isopeptide linked E2~Ub conjugate and the reactive thioester linked E2~Ub conjugate at 22 °C. Gaussians are fitted to the low FRET state (green), the intermediate FRET state (red) and the high FRET state (blue). The multiple Gaussian fit is shown in black. The pie charts to the right of each histogram show the percentage contribution of each FRET state to the overall population, using the same colour scheme as the Gaussians fitted to the smFRET histograms. **b** Normalized smFRET histogram showing conformation

of the thioester linked E2~Ub conjugate at 22 °C. The histogram is outlined in blue. Gaussians are fitted to the low FRET state (green), the intermediate FRET state (red) and the high FRET state (dark blue). The multiple Gaussian fit is shown in black. Source data are provided as a Source Data file.

Supplementary Figure 13

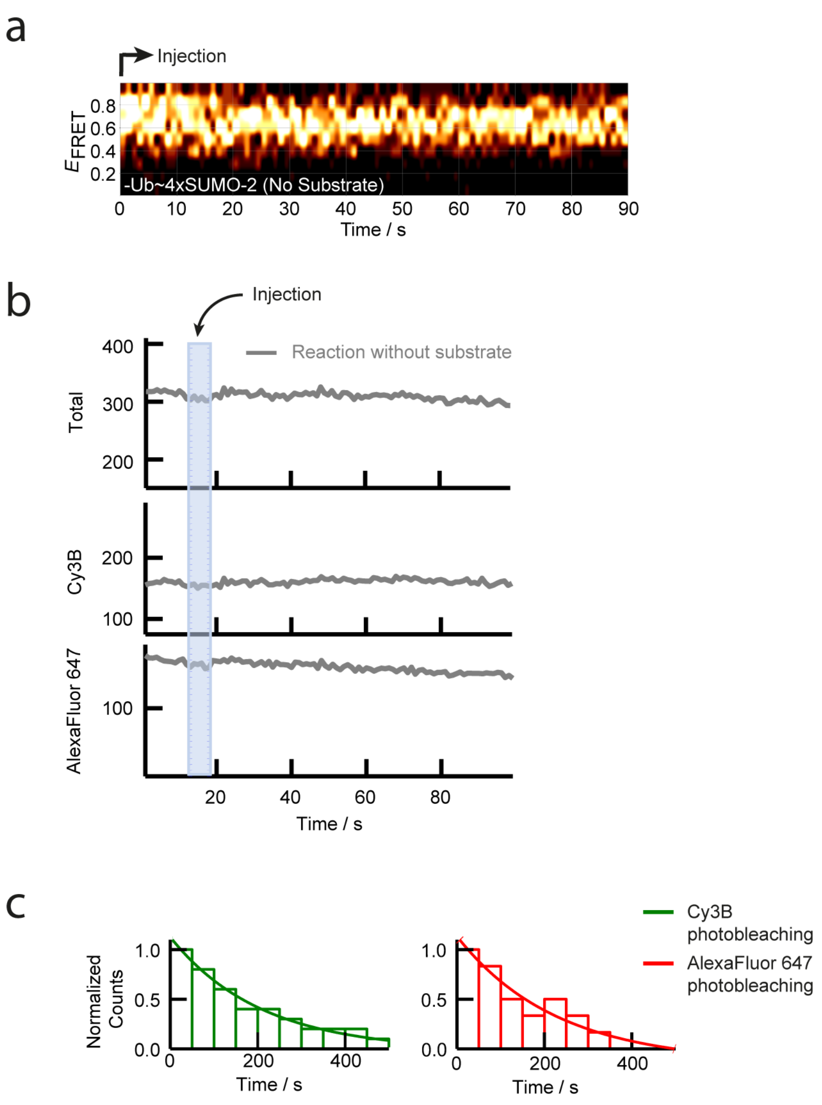

Supplementary Fig. 13 Analysis of real-time smFRET reaction in the absence of substrate. **a** Single molecule contour plot showing the progression of the FRET trajectory following the real-time injection of UEV and RNF4^{WT} in the absence of substrate. **b** Cumulative intensity variation (grey) from all fluorescent spots over time for the injection shown in **a**. The total fluorescence intensity is shown along with separate Cy3B and AlexaFluor 647 intensities, with the injection interval highlighted in blue. **c** smFRET dwell-time histograms obtained for Cy3B photobleaching (green,

left panel) and Alexa647 photobleaching (red, right panel) for the reaction shown in **a**. The solid lines represent the fit to single exponential decay functions. Source data are provided as a Source Data file.

Supplementary Figure 14

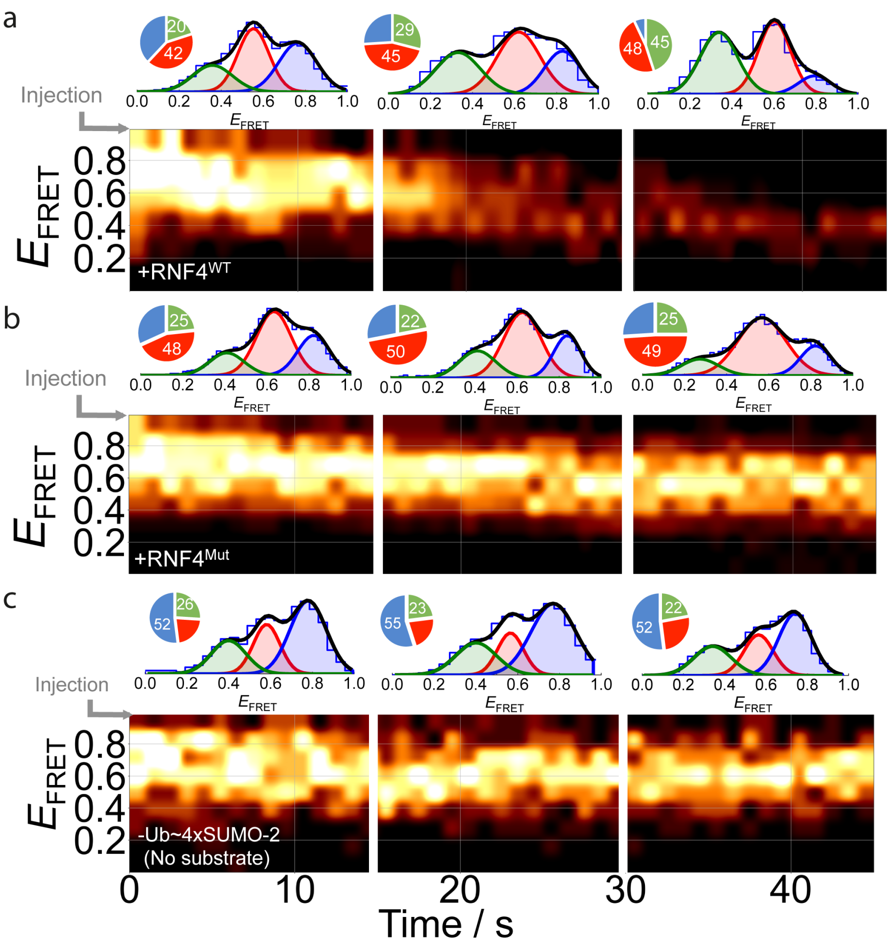

Supplementary Fig. 14 Analysis of FRET states of the real-time smFRET reaction. **a** Single molecule contour plot showing the progression of the FRET trajectory for all FRET containing molecules following the real-time injection of UEV, RNF4^{WT} and Ub~4xSUMO-2 (substrate) split into 15 second intervals. Normalized smFRET histograms generated from each 15-second interval are shown above the contour plot. The histogram is outlined in blue. Gaussians are fitted to the low FRET state (green), the intermediate FRET state (red) and the high FRET state (dark blue). The multiple Gaussian fit is shown in black. Pie charts to the left of each histogram highlight the percentage contribution of each FRET state to the overall population, using the same

colour scheme as the Gaussians fitted to the smFRET histograms. **b** Same as in **a** but for the RNF4^{MUT} version. **c** Same as in **a** but for the reaction in the absence of substrate. Source data are provided as a Source Data file.

Supplementary Figure 15

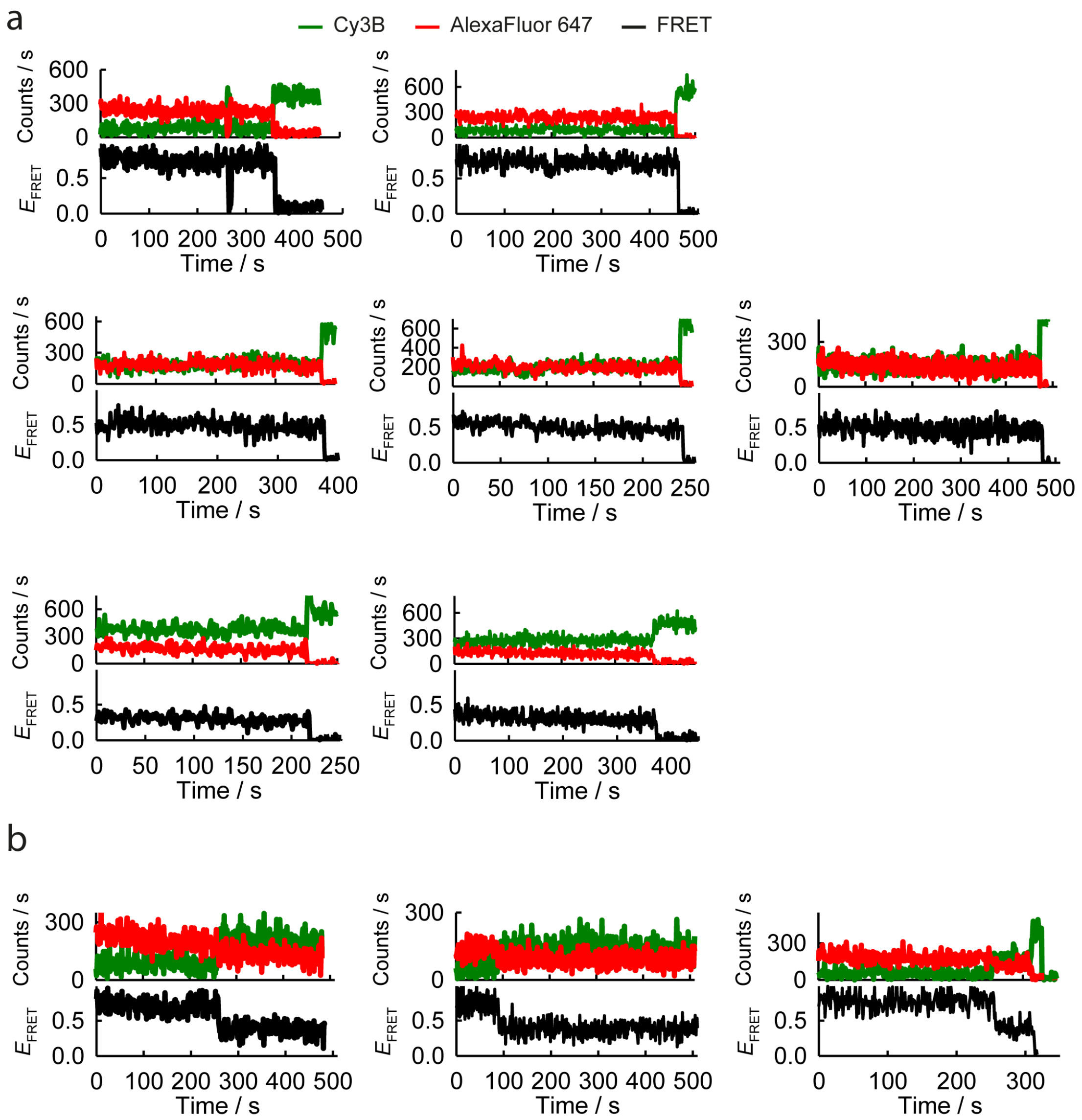

Supplementary Fig. 15 Representative single molecule traces. **a** Representative single molecule traces for the real-time reaction with RNF4^{MUT} demonstrating the stability for the long duration of the reaction. Each molecule contains a Cy3B (green), AlexaFluor 647 (red) and FRET intensity trace (black). **b** Same as in **a** but demonstrating rare interconversion events to lower FRET states. Source data are provided as a Source Data file.
