# Supplementary material for: Ubiquitin transfer by a RING E3 ligase occurs from a closed E2~ubiquitin conformation
Source: Nat Commun. 2020 Jun 5;11:2846. doi: 10.1038/s41467-020-16666-y (PMC7275055; doi:10.1038/s41467-020-16666-y)
Supplement: Supplementary file 3 — Reporting Summary [file 41467_2020_16666_MOESM3_ESM.pdf]

nature research

Corresponding author(s): Ronald T. Hay and J. Carlos Penedo

Last updated by author(s): May 7, 2020

Reporting Summary

Nature Research wishes to improve the reproducibility of the work that we publish. This form provides structure for consistency and transparency in reporting. For further information on Nature Research policies, see Authors & Referees and the Editorial Policy Checklist.

Statistics

For all statistical analyses, confirm that the following items are present in the figure legend, table legend, main text, or Methods section.

n/a	Confirmed	
☐	☒	The exact sample size (*n*) for each experimental group/condition, given as a discrete number and unit of measurement
☐	☒	A statement on whether measurements were taken from distinct samples or whether the same sample was measured repeatedly
☒	☐	The statistical test(s) used AND whether they are one- or two-sided<br>*Only common tests should be described solely by name; describe more complex techniques in the Methods section.*
☒	☐	A description of all covariates tested
☒	☐	A description of any assumptions or corrections, such as tests of normality and adjustment for multiple comparisons
☐	☒	A full description of the statistical parameters including central tendency (e.g. means) or other basic estimates (e.g. regression coefficient) AND variation (e.g. standard deviation) or associated estimates of uncertainty (e.g. confidence intervals)
☒	☐	For null hypothesis testing, the test statistic (e.g. *F*, *t*, *r*) with confidence intervals, effect sizes, degrees of freedom and *P* value noted<br>*Give P values as exact values whenever suitable.*
☒	☐	For Bayesian analysis, information on the choice of priors and Markov chain Monte Carlo settings
☒	☐	For hierarchical and complex designs, identification of the appropriate level for tests and full reporting of outcomes
☒	☐	Estimates of effect sizes (e.g. Cohen's *d*, Pearson's *r*), indicating how they were calculated

Our web collection on statistics for biologists contains articles on many of the points above.

Software and code

Policy information about availability of computer code

Data collection	IDL 6.0 was used to process data.
Data analysis	Data were analysed using routines written in Matlab, which are described in McCluskey, K., Shaw, E., Lafontaine, D. A. & Penedo, J. C. in Methods in Molecular Biology Vol. 1076 (eds Y. Engelborghs & A. J. W. G. Visser) 759-791 (Humana Press, 2014).

For manuscripts utilizing custom algorithms or software that are central to the research but not yet described in published literature, software must be made available to editors/reviewers. We strongly encourage code deposition in a community repository (e.g. GitHub). See the Nature Research guidelines for submitting code & software for further information.

Data

Policy information about availability of data

All manuscripts must include a data availability statement. This statement should provide the following information, where applicable:

- Accession codes, unique identifiers, or web links for publicly available datasets
- A list of figures that have associated raw data
- A description of any restrictions on data availability

Data underlying all Figures and Supplementary Figures are available in a source data file at https://doi.org/10.17630/5f652bc1-1d66-400a-bd76-609b7de0bb25.

Field-specific reporting

Please select the one below that is the best fit for your research. If you are not sure, read the appropriate sections before making your selection.

☒ Life sciences ☐ Behavioural & social sciences ☐ Ecological, evolutionary & environmental sciences

Life sciences study design

All studies must disclose on these points even when the disclosure is negative.

Sample size	FRET population in single molecule histograms were built from hundreds of molecules and their relative contributions, fitted using gaussian functions, were considered statistically significant when increasing the number of molecules did not alter the centre and width of each gaussian.
Data exclusions	Single molecule FRET traces were excluded if they did not adhere to the characteristics of a single molecule (i.e. signal intensity, donor and acceptor anti-correlation and single photobleaching event for each fluorescent dye).
Replication	All replication attempts were successful. For single molecule FRET experiments, histograms were generated from movies throughout the channel and between different channels and between channels on different slides to determine reproducibility.
Randomization	This is not applicable to this study as it is not a placebo controlled trial.
Blinding	This is not applicable to this study as it is not a placebo controlled trial.

Reporting for specific materials, systems and methods

We require information from authors about some types of materials, experimental systems and methods used in many studies. Here, indicate whether each material, system or method listed is relevant to your study. If you are not sure if a list item applies to your research, read the appropriate section before selecting a response.

Materials & experimental systems

n/a	Involved in the study	
☐	☒	Antibodies
☒	☐	Eukaryotic cell lines
☒	☐	Palaeontology
☒	☐	Animals and other organisms
☒	☐	Human research participants
☒	☐	Clinical data

Methods

n/a	Involved in the study	
☒	☐	ChIP-seq
☒	☐	Flow cytometry
☒	☐	MRI-based neuroimaging

Antibodies

Antibodies used	Anti-ubiquitin primary antibody (Dilution 1:2000, Dako, Z0458)<br>Anti-rabbit IgG (whole molecule) Peroxidase secondary antibody (Dilution 1:10000, Sigma, A6154)
Validation	All antibodies used in this study are commercially available. Antibodies have been validated by the manufactures and used in the following previous studies:<br>1. Plechanovová, A., Jaffray, E. G., Tatham, M. H., Naismith, J. H. & Hay, R. T. Structure of a RING E3 ligase and ubiquitin-loaded E2 primed for catalysis. Nature 489, 115-120, doi:10.1038/nature11376 (2012)<br>2. Branigan, E., Plechanovová, A., Jaffray, E. G., Naismith, J. H. & Hay, R. T. Structural basis for the RING-catalyzed synthesis of K63-linked ubiquitin chains. Nature Structural & Molecular Biology 22, 597-602, doi:10.1038/nsmb.3052 (2015)).
