# Supplementary material for: Ubiquitin transfer by a RING E3 ligase occurs from a closed E2~ubiquitin conformation
Source: Nat Commun. 2020 Jun 5;11:2846. doi: 10.1038/s41467-020-16666-y (PMC7275055; doi:10.1038/s41467-020-16666-y)
Supplement: Supplementary file 4 — Source Data [file 41467_2020_16666_MOESM4_ESM.zip › Source_Data/Code/Python scripts.docx]

**** Python Scripts for contour plot generation from FRET trajectories in ASCII format**

**--CODE STARTS HERE--**

# -*- coding: utf-8 -*-

"""

Created on Mon Sep 2 14:34:19 2019

Contour plot fret traces

@author: carlos penedo

"""

import os

import numpy as np

import matplotlib.pyplot as plt

import pandas as pd

folder_name = r'C:\papers\published 2019\Branigan et al 2019_DONE\data\sigma plot files\Figure 4\ASCII_44' (**Replace this folder with the location folder for the data to be analyzed)

f_names = [f for f in os.listdir(folder_name) if f.endswith(r'.dat')]

full_names = [os.path.join(folder_name, f) for f in f_names]

names = ['time','donor','acc','fret']

data = [pd.read_csv(f, header=-1, delim_whitespace=True, names=names) for f in full_names]

for trace in data:

idx_don = (trace['donor']<7).idxmax()

idx_acc = (trace['acc']<7).idxmax()

idx = min([idx_don, idx_acc])

trace['fret'][idx:] = np.nan

arr = np.stack(i['fret'] for i in data)

ini_, fin_, delta_bin = 0.0, 0.99, 0.095

bins = np.arange(ini_, fin_, delta_bin)

img = []

for col in arr.T:

img.append(np.histogram(col, bins=bins)[0])

img = np.stack(img)

num_t_points = 90 # or x-axis limit

integration_t = 1 # 100 ms?

plt.imshow(np.flipud(img.T)[:,:num_t_points], aspect='auto',vmin=0.5,vmax=15,

interpolation='kaiser',cmap='afmhot',

extent=[0, num_t_points*integration_t, ini_, fin_])

ax = plt.gca()

ax.set_xticks(np.arange(0,90,10))

ax.set_yticks([0.2,0.4,0.6,0.8])

ax.set_position([0.1,0.3,0.7,0.3])

plt.ylabel('')

plt.xlabel('')

#ax.set_xticklabels([])

plt.grid(True)

def adjust_fontsize():

FONTSIZE = 100

list_font = ([ax.title, ax.xaxis.label, ax.yaxis.label] +

ax.get_xticklabels() + ax.get_yticklabels())

[it.set_fontsize(FONTSIZE) for it in list_font]

[it.set_fontname('arial') for it in list_font]

adjust_fontsize()

**--CODE ENDS HERE--**

**** Python Scripts for 3D plots of single molecule histograms**

**--CODE STARTS HERE--**

# -*- coding: utf-8 -*-

"""

Created on Tue Jul 23 11:28:54 2019

@author: Carlos Penedo

"""

import os

import numpy as np

import pandas as pd

from mpl_toolkits.mplot3d import Axes3D as axes3D

def read_data():

folder_name = r'C:\papers\published 2019\Branigan et al 2019_DONE\data\sigma plot files\Figure 1\ASCII'

f_names = [f for f in os.listdir(folder_name) if f.endswith(r'.CSV')]

full_names = [os.path.join(folder_name, f) for f in f_names]

col_names = ['FRET1', 'cts_norm', 'FRET2', 'gauss1', 'gauss2','gauss2',

'gaus1', 'gaus2', 'gaus_sum']

data = [pd.read_csv(f, names=col_names, header=0) for f in full_names]

return data

def make_plot():

y_ticks = np.arange(len(data))+0.1

x_ticks = np.arange(0, 1.1, 0.2)

fig = plt.figure()

ax = fig.add_subplot(111, projection='3d')

for yy, dd in zip(y_ticks, data):

# bar

x_bar = dd['FRET'].dropna().values

y_bar = dd['cts_norm'].dropna().values

ax.bar(x_bar, y_bar, zs=yy, zdir='y', width=-0.03, align='edge',

color='r', alpha=0.5, edgecolor=[0]*3, linewidth=1, zorder=2)

# fit line

xx = dd['FRET_xx'].dropna().values

y_fit = dd['gaus_sum'].dropna().values[:len(xx)]

ax.plot(xx, y_fit, zs=yy-0.01, zdir='y', color='k', lw=2, zorder=1)

for x_ti in x_ticks:

# grid line

y_grid = [-0.1, 7.1] # always the same

x_grid = [x_ti]*2

ax.plot(x_grid, y_grid, zs=0, zdir='z', color=[0.25]*3, ls='--', lw=1)

return fig, ax

def tweak_plot():

ax.w_zaxis.line.set_lw(0.)

ax.set_zticks([])

# make the panes transparent

ax.xaxis.set_pane_color((1.0, 1.0, 1.0, 0.0))

ax.yaxis.set_pane_color((1.0, 1.0, 1.0, 0.0))

ax.zaxis.set_pane_color((1.0, 1.0, 1.0, 0.0))

# make the grid lines transparent

ax.xaxis._axinfo["grid"]['color'] = (1,1,1,0)

ax.yaxis._axinfo["grid"]['color'] = (1,1,1,0)

ax.zaxis._axinfo["grid"]['color'] = (1,1,1,0)

ax.set_xlim([0, 1.0])

ax.set_ylim([0, 7.2])

ax.set_zlim([0, 0.2])

ax.invert_xaxis()

y_ticks_labels = []

ax.set_yticklabels(y_ticks_labels)

plt.xlabel('FRET efficiency', labelpad=20)

def stretch_y():

x_scale = 1

y_scale = 2

z_scale = 1

scale=np.diag([x_scale, y_scale, z_scale, 1.0])

scale=scale*(1.0/scale.max())

scale[3,3]=1.0

def short_proj():

return np.dot(axes3D.get_proj(ax), scale)

ax.get_proj=short_proj

def adjust_fontsz():

FONTSIZE = 12

list_font = ([ax.title, ax.xaxis.label, ax.yaxis.label] +

ax.get_xticklabels() + ax.get_yticklabels())

[it.set_fontsize(FONTSIZE) for it in list_font]

[it.set_fontname('cambriai') for it in list_font]

def final_tweak():

# ax.view_init(elev=145, azim=45)

plt.setp( ax.yaxis.get_majorticklabels(), ha="left", rotation=-20 );

data = read_data()

fig, ax = make_plot()

tweak_plot()

stretch_y()

final_tweak()

adjust_fontsz()

**--CODE ENDS HERE--**
