# Supplementary figures and images for: Ubiquitin transfer by a RING E3 ligase occurs from a closed E2~ubiquitin conformation
Source: Nat Commun. 2020 Jun 5;11:2846. doi: 10.1038/s41467-020-16666-y (PMC7275055; doi:10.1038/s41467-020-16666-y)

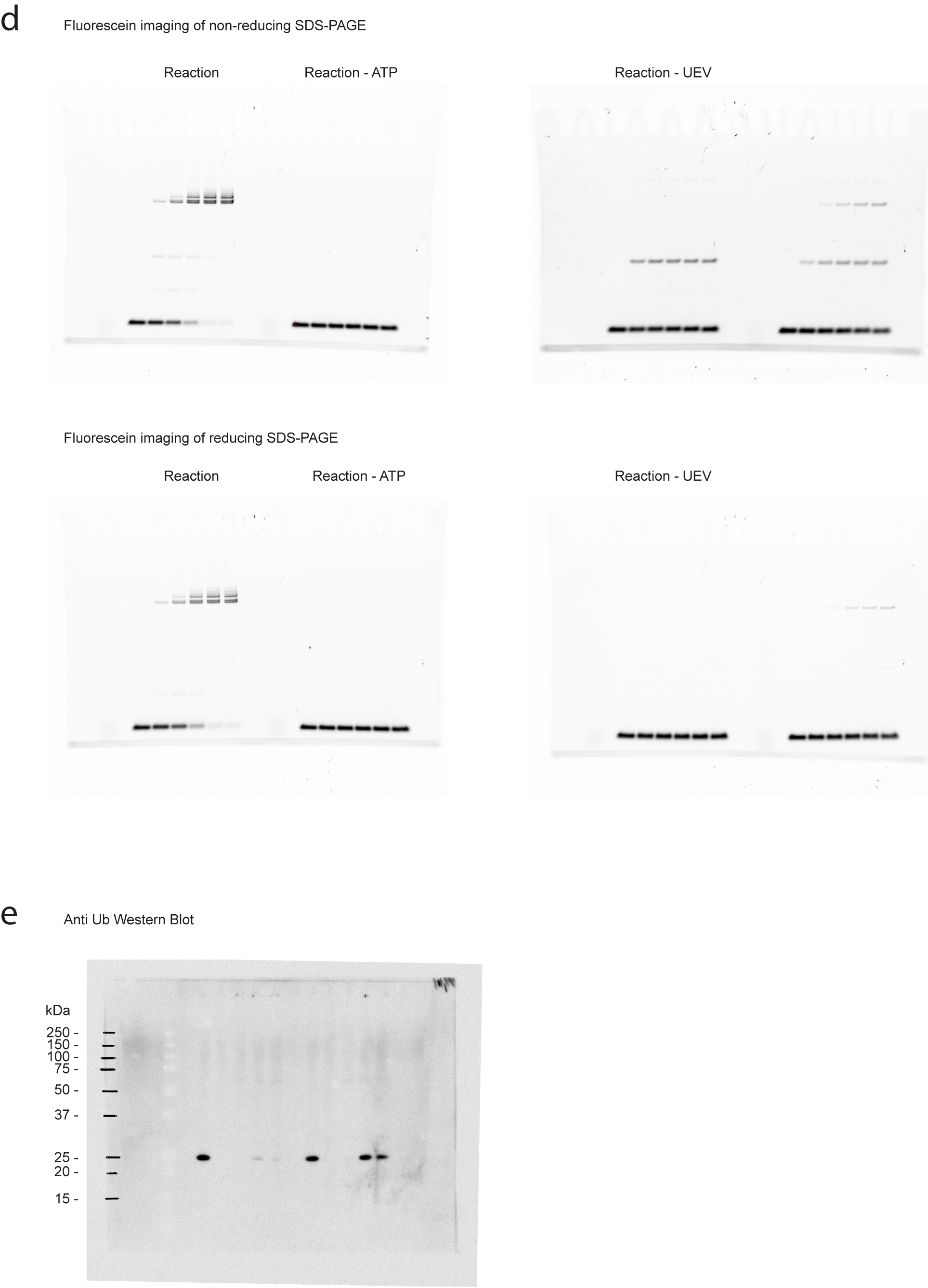

Supplement: Supplementary file 4 — Source Data [file 41467_2020_16666_MOESM4_ESM.zip › Source_Data/Figures/Figure_1/Figure_1d_and_e.tif]
